# The normal trachea is cleaned by MUC5B mucin bundles from the submucosal glands coated with the MUC5AC mucin

**DOI:** 10.1016/j.bbrc.2017.08.113

**Published:** 2017-10-21

**Authors:** Anna Ermund, Lauren N. Meiss, Ana M. Rodriguez-Pineiro, Andrea Bähr, Harriet E. Nilsson, Sergio Trillo-Muyo, Caroline Ridley, David J. Thornton, Jeffrey J. Wine, Hans Hebert, Nikolai Klymiuk, Gunnar C. Hansson

**Affiliations:** a Department of Medical Biochemistry, University of Gothenburg, SE-405 30 Gothenburg, Sweden; b Institute of Molecular Animal Breeding and Biotechnology, Gene Center, Ludwig-Maximilians-University Munich, Feodor-Lynen-Straße 25, 81377 Munich, Germany; c Department of Biosciences and Nutrition, Karolinska Institutet, and School of Technology and Health, KTH Royal Institute of Technology, Novum, SE-141 57 Huddinge, Sweden; d Wellcome Trust Centre for Cell-Matrix Research, Faculty of Biology, Medicine and Health, University of Manchester, Manchester, UK; e The Cystic Fibrosis Research Laboratory, Stanford University, Stanford, CA 94305, United States

**Keywords:** Respiratory tract, Mucus, MUC5AC, Airway surface liquid, Lung

## Abstract

To understand the mucociliary clearance system, mucins were visualized by light, confocal and electron microscopy, and mucus was stained by Alcian blue and tracked by video microscopy on tracheal explants of newborn piglets. We observed long linear mucus bundles that appeared at the submucosal gland openings and were transported cephalically. The mucus bundles were shown by mass spectrometry and immunostaining to have a core made of MUC5B mucin and were coated with MUC5AC mucin produced by surface goblet cells. The transport speed of the bundles was slower than the airway surface liquid flow. We suggest that the goblet cell MUC5AC mucin anchors the mucus bundles and thus controls their transport. Normal clearance of the respiratory tree of pigs and humans, both rich in submucosal glands, is performed by thick and long mucus bundles.

## Highlights

• Submucosal glands in the piglet trachea form bundles of MUC5B mucin.
• The mucus bundles are coated with MUC5AC mucin produced by surface goblet cells.
• The mucus bundles are transported 10-times slower than the airway surface liquid.
• The surface goblet cells are suggested to control the mucus bundle movement.

## 1 Introduction

The respiratory system is kept relatively free from inhaled bacteria and debris by the mucociliary clearance system. The cilia generate the movement by their continuous beating [11]. The major constituents in the mammalian mucus are the MUC5B and the MUC5AC mucins. The MUC5B mucin is normally made by submucosal glands and the MUC5AC mucin by surface goblet cells. These two mucins have similar domain organization, and both form disulfide-bonded dimers by their *C*-termini [14]. MUC5B is arranged as linear molecules, similarly to the related von Willebrand factor (VWF), as it forms disulfide-bonded dimers in its *N*-terminus [9]. The properties of these secreted mucins are highly influenced by the local conditions at release from the mucin-producing cells, as shown for MUC2 in the intestine [4].

The different organization of glands and goblet cells in rodents, pigs and humans suggests differences in how the mucociliary clearance system functions. In contrast to mice, the pig system is similar to the one in humans. Piglets have been used for *in vivo* studies of gross mucociliary clearance using single-particle tantalum microdiscs, showing migration cephalically and from dorsal to ventral side [6]. Strands, suggested to contain mucins, were also observed at the submucosal gland openings, but their relation to the clearance of fluorescent nanospheres (beads) has not been clarified [7]. In a preliminary report we established methods to visualize the mucus bundles and preliminary defined their composition [5]. We now show that submucosal glands secrete MUC5B mucin molecules that form linear polymers and are coated by the MUC5AC mucin. These bundles are transported slower and separately from the airway surface liquid (ASL) mass flow.

## 2 Material and methods

### 2.1 Piglet airway preparation and staining

Piglets were euthanized under Ketamine (Ursotamin^®^, Serumwerk Bernburg, Germany) and Azaperone (Stresnil^®^, Elanco Animal Health, Bad Homburg, Germany) anesthesia by intracardial injection of T61^®^ (Intervet, Unterschleissheim, Germany). Airways including the larynx, trachea and lungs were explanted and immersed in chilled Perfadex^®^ solution (XVIVO Perfusion, Gothenburg, Sweden) adjusted to pH 7.2 with 1 M TBS. All connective and pulmonary tissue was removed and the prepared airways transferred to a 50 ml tube with Perfadex^®^ solution before shipping under chilled conditions overnight to Gothenburg. Ethical permissions for the pig experiments were obtained from Regierungen von Oberbauern, Münich, Germany and Jordbruksverket, Jönköping, Sweden.

The distal trachea and proximal part of the primary bronchi were mounted in a Petri dish coated with Sylgard 184 Silicone Elastomer (Dow Corning, Wiesbaden, Germany) using 27G needles. The tissue was covered in oxygenated (95% O_2_, 5% CO_2_) Krebs-glucose buffer in 116 mM NaCl, 1.3 mM CaCl_2_, 3.6 mM KCl, 1.4 mM KH_2_PO_4_, 23 mM NaHCO_3_, and 1.2 mM MgSO_4_, 10 mM d-glucose, 5.7 mM pyruvate, 5.1 mM glutamate, pH 7.4, and gradually heated to 37 °C. Tissues were stained with 0.4 mM Alcian blue 8GX, charcoal [4] and/or with 40 nm carboxylate-modified fluorescent (580/605) microspheres (FluoSpheres, Thermo), hereafter called “beads”. Tissue was monitored through a stereo microscope with color or monochrome CCD cameras (DS-Fi2 or DS-QiMc, Nikon). The speed of the Alcian blue-stained bundles (mean of five measurements in each time-lapse), and thickness were calculated using NIS elements (Nikon). Bundle movement patterns were calculated by selecting five evenly distributed points on one bundle.

### 2.2 Tissue imaging

Pig airway tissue (4% formalin fixed) was stained by *anti*-MUC5AC (45M1, Sigma), and *anti*-MUC5B (kind gift from M. Kesimer, University of North Carolina Chapel Hill, NC) and nuclei were counterstained with Hoechst 34580 (Thermo).

Live pig airway tissue was opened, glued to a Petri dish (Vetbond, 3 M), and visualized with fluorescein labeled *Lotus tetragonolobus* lectin (LTL, Vector Laboratories), rhodamine labeled *Ulex europaeus* agglutinin I (UEAI, Vector Laboratories) and 40 nm carboxylate-modified fluorescent (580/605) beads. Tissues were imaged with a Plan-Apochromat 20×/1.0 DIC objective in a LSM 700 Axio confocal microscope (Carl Zeiss) and analyzed using Imaris (version 7.6.3, Bitplane, Zürich, Switzerland).

### 2.3 Scanning electron microscopy (SEM)

Pig airway tissue was fixed in Karnovsky's fixative for 24 h followed by postfixation in 1% OsO_4_ at 4 °C for 3 times intervened by 1% thiocarbohydrazide. Ethanol dehydrated samples were incubated with hexamethyldisilazane, sputter-coated with palladium and analyzed by SEM in a DSM 982 Gemini (Carl Zeiss).

### 2.4 Gel electrophoresis

Samples were washed twice in 10 mM Tris buffer pH 7.5, passed through a 21G needle, diluted in loading buffer, loaded onto 3–8% NuPAGE Tris-Acetate gels, and separated in Tris-Acetate buffer, blotted to a PVDF membrane (Immobilon-P 0.45 μm, Millipore), and stained with 0.125% Alcian blue or with *anti*-MUC5B antibody.

### 2.5 Proteomic analysis

Whole mucus and individual Alcian blue-stained mucus bundles were subjected to relative quantification of their total protein [10]. Assembled pig MUC5B and MUC5AC sequences at www.medkem.gu.se/mucinbiology/databases were used to select labeled peptides; MUC5B: TQAACPNAK, TSVHIQLHQR, LTPLQFGNLQK, FSLEAPAVQCR, TVTLSLNGGDTAIR and LVQALGAGGCCPTFR; MUC5AC: CGCVGQR, SSVLVNGR, LTDTHGPFAR, LSPIEFGNLQK and NATPGATGAGCQK. 300 fmol of the peptides (SpikeTides TQL, JPT Peptide Technologies) were added and quantified with Skyline v. 3.5.

### 2.6 Statistical analysis

Statistical tests were performed using GraphPad Prism 6.07 (GraphPad), and data represented as standard error of the mean. Each time-lapse was considered as one replicate. Differences were assessed with the Mann-Whitney *U* test to compare two groups and with the Kruskal-Wallis test followed by Sidak correction for multiple groups. *P* < 0.05 was defined as significant.

## 3 Results

### 3.1 Mucus bundles secreted from the pig submucosal glands

**Fig. 1:**
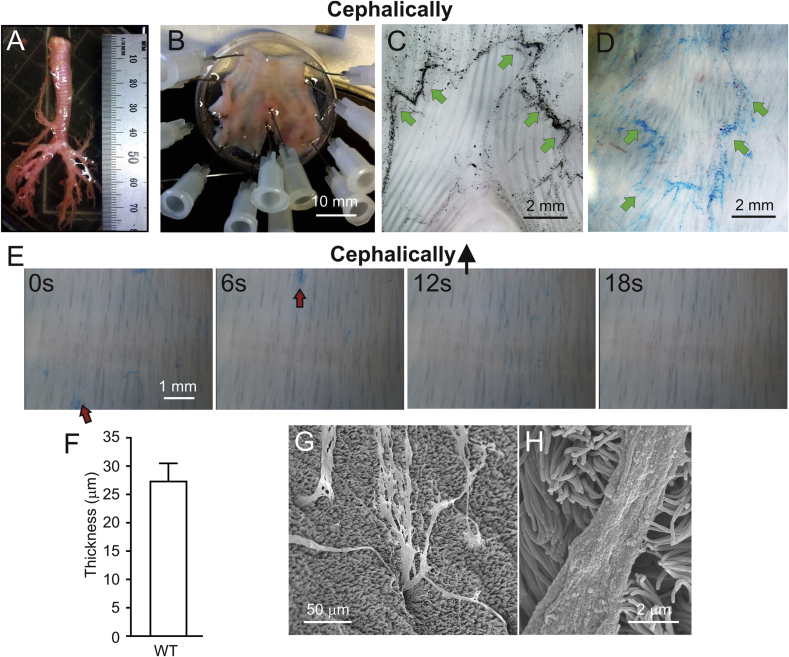
**Alcian blue-stained bundles from piglet submucosal glands are transported cephalically**. A. Pig trachea. B. Distal trachea and primary bronchi mounted in the experimental chamber. C. Dorsal side of piglet trachea with bifurcation and charcoal collected in the mucus. Mucus was moved cephalically and outwards as indicated by the green arrows. D. The ventral side of the trachea with Alcian blue-stained bundles moving according to the arrows, up and ventrally. E. Pig trachea mounted as in B with Alcian blue-stained bundles moving upwards over the surface in Movie S1. Images at 0, 6, 12 and 18 s from the same surface of a time-lapse recording at 16 times the original speed. Red arrows point to the same mucus bundles. F. Diameter of Alcian blue-stained bundles in airways, mean of 8 bundles from 3 pigs. G. SEM image of a pig trachea with bundles coming out of submucosal glands. H. Mucus bundle and cilia appear to interact.

As pigs have numerous submucosal glands similarly to humans, we have used their tracheobronchial tree as a model for normal mucus transport (Fig. 1A). The trachea and primary bronchi were opened from either the ventral or dorsal side and then mounted (Fig. 1B). We have previously used charcoal as a way to visualize intestinal mucus [4] and when added to the dorsal side these particles were moved cephalically and laterally (Fig. 1C). As the charcoal binding to mucus is unspecific, we instead turned to the positively charged Alcian blue staining that relatively specifically binds the densely negatively charged lung mucins. Alcian blue-stained ventral trachea revealed how bundles moved cephalically and were collected ventrally (Fig. 1D), similarly to what has been observed for tantalum microdiscs *in vivo* [6].

The Alcian blue-stained bundles moved with varying speed and intermittently, as shown in Fig. 1E, and Movie S1. The average speed of the bundles was calculated to be 0.34 ± 0.1 mm/min. The mucus bundles on piglet explants had a diameter of around 27 μm (Fig. 1F). The individual bundles sometimes became entangled with each other and had a tendency to gather into even thicker bundles. The origin of the bundles was suggested to be the submucosal glands as SEM showed long continuous bundles coming out of submucosal gland openings (Fig. 1G). Once out on the tracheobronchial surface, the mucus bundles appeared to interact with the cilia as shown by SEM (Fig. 1H). Mucus bundles appearing at gland openings have previously been observed in humans and piglets [7], [15].

Supplementary video related to this article can be found at http://dx.doi.org/10.1016/j.bbrc.2017.08.113.

The following is the supplementary data related to this article:

Movie S1 Video of Alcian blue-stained mucus bundle movement. Movie S1

### 3.2 The mucus bundles contain the MUC5B mucin

**Fig. 2:**
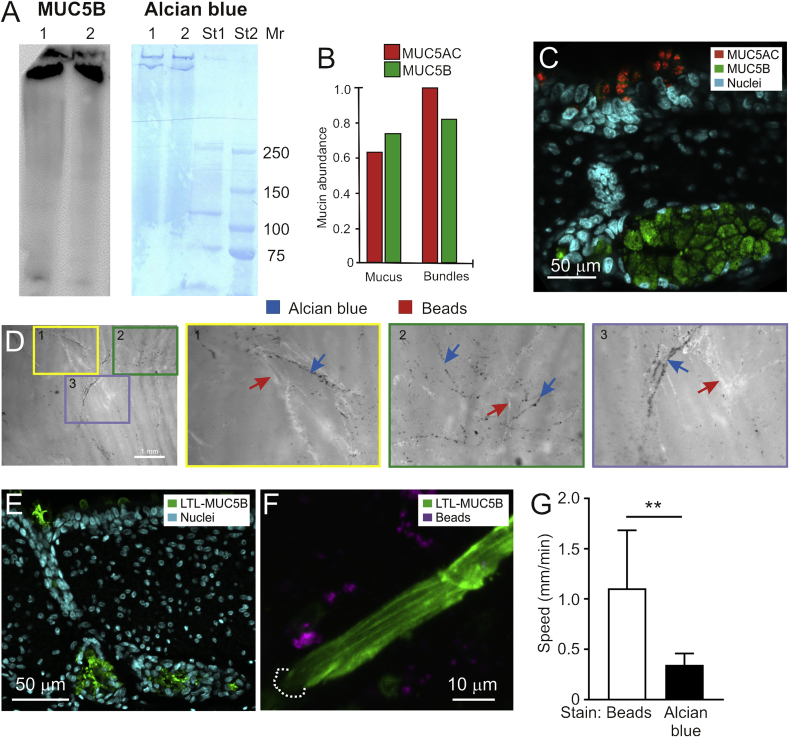
**The piglet MUC5B mucin bundles are stained by Alcian blue but not fluorescent beads.** A. SDS-PAGE and Western blot stained with *anti*-MUC5B (left) and gel directly stained with Alcian blue (right). Lane 1 and 2 were loaded with gently scraped mucus from piglet trachea. Protein standards (St1, St2) with molecular mass (Mr, kDa) of St2. Representative of three repeats. B. Relative abundance of MUC5AC and MUC5B in mucus and bundles by targeted proteomics and relative to MUC5AC in bundles. C. Immunofluorescence of pig trachea. MUC5AC in the surface goblet cells (red) and MUC5B in the submucosal gland (green). D. Mounted piglet tracheas stained with Alcian blue and 40 nm fluorescent beads. Three colored areas were magnified. The 40 nm fluorescent beads (white material, marked with red arrows) do not stain the same structures as Alcian blue (black material, marked by blue arrows). Identical results were obtained for 10 pigs. E. LTL lectin (green) stains submucosal gland mucins (MUC5B). F. Live trachea with mucus bundles stained with the LTL lectin for MUC5B (green) and added 40 nm fluorescent beads (purple) showing little colocalization. The experiment was repeated 3 times. G. The mean speed of mucus bundles stained with Alcian blue or 40 nm fluorescent beads, n = 8, **p = 0.002.

The surface of piglet trachea was gently scraped to obtain a whole mucus fraction which was separated by gel electrophoresis and blotted. MUC5B immunostaining revealed staining at the very top of the gel (Fig. 2A). When adjacent lanes on the blot were stained with Alcian blue, the same bands as for MUC5B were observed. No smaller bands were detected, suggesting that the mucins were the only molecules in the sample stained by Alcian blue.

To analyze the molecular nature of the mucus bundles, the surface of piglet trachea was gently scraped to obtain a whole mucus fraction. Alcian blue-stained mucus bundles were collected separately. These samples were subjected to proteomic analyses. Since the publically available pig databases were poorly assembled for the *Muc5b* and *Muc5ac* genes, more accurate sequences were assembled by cDNA sequencing (www.medkem.gu.se/mucinbiology/databases). Due to the high sequence homology, MUC5B and MUC5AC were summed for the complete proteome (Table 1 and Table S1). Major secreted proteins identified by proteomic analyses are presented in Table 1 (Table S1 for the complete proteomics results). Among the proteins observed were known lung proteins such as surfactants. Interestingly, collected mucus bundles showed similar protein composition to the total mucus. Next, the MUC5B and MUC5AC mucins were specifically quantified by targeted mass spectrometry, showing a MUC5AC/MUC5B ratio of 0.9 in whole mucus and slight relative increase of MUC5AC in the collected bundles (Fig. 2B). Analysis of the cellular origin of the mucins by immunostaining showed that submucosal glands were only stained for MUC5B, whereas the surface goblet cells were stained for the MUC5AC mucin (Fig. 2C).

**Table 1:** Summary of the relative abundance (ppm) of selected secreted proteins from whole mucus and individual mucus bundles. For the complete dataset, see Table S1.

Gene name	Protein name	Topology prediction^a^	Whole mucus (ppm; n = 4)	Mucus bundles (ppm; n = 10)	Pep-tides	Unique peptides	UniProt identifier
*Muc5ac* + *Muc5b*^b^	Mucin-5ac + Mucin-5b	SEC	1063	744	41	41	–
*Afp*	Alpha-fetoprotein	SEC	1642	2037	48	48	Q8MJ76
*Agr2*	Anterior gradient protein 2 homolog	SEC	4567	4529	15	15	I3LLU1
*Ahsg*	Alpha-2-HS-glycoprotein	SEC	11298	12024	16	16	F1SFI7
*Alb*	Serum albumin	SEC	13074	18501	55	40	F1RUN2
*Apoa1*	Apolipoprotein A-I	SEC	3444	4148	32	31	A0A0C3SG01
*Calr*	Calreticulin	SEC	1456	1111	27	27	P28491
*Clca1*	Calcium-activated chloride channel regulator 1	SEC	2608	2548	44	11	F1S4C9
*Ctsb*	Cathepsin B	SEC	37	12	5	5	A1E295
*Ctsl*	Cathepsin L1	SEC	6	0.0	2	2	Q28944
*Fcgbp*	IgGFc-binding protein	SEC	20	12	6	6	I3LT38
*Grp-58*	Protein disulfide-isomerase	SEC	4597	3264	39	39	E1CAJ5
*Hspa5*	78 kDa glucose-regulated protein	SEC	5372	4399	41	40	F1RS36
*Orm1*	Alpha-1-acid glycoprotein	SEC	23207	26258	18	18	F1SN68
*Pdia4*	Protein disulfide-isomerase	SEC	2413	1831	34	34	F1SAD9
*Psap*	Saposin-B-Val	SEC	121	116	7	6	F1SU97
*Scgb3a1*	Secretoglobin family 3A member 1	SEC	0	36	5	5	F1S5Q5
*Serpina1*	Alpha-1-antitrypsin	SEC	890	1004	23	23	F1SCF0
*Serpina3-2*	Alpha-1-antichymotrypsin 2	SEC	16	5	13	9	Q9GMA6
*Serpina3-3*	Serpin A3-3	SEC	1439	1776	17	10	F1SCD0
*Serpina6*	Corticosteroid-binding globulin	SEC	7	7	3	3	Q9GK37
*Serpinc1*	Antithrombin-III	SEC	382	265	26	25	F2Z5E2
*Serping1*	Plasma protease C1 inhibitor	SEC	5	4	3	3	F1SJW8
*Sftpa1*	Pulmonary surfactant-associated protein A	SEC	8	10	5	5	F1SER3
*Sftpb*	Pulmonary surfactant-associated protein B	SEC	0	1	2	2	F1SVC0

a **SEC:** predicted to be secreted.

b The relative amount of the MUC5AC and MUC5B mucins could be underestimated due to the heavy protein glycosylation.

Others have used fluorescent 40 nm beads (nanospheres) for staining mucus [7]. These negatively charged beads are not expected to stain the negatively charged mucins, as repulsion forces should keep them apart. When tracheal tissue was co-stained with these fluorescent beads (Fig. 2D, white staining marked with red arrows) and Alcian blue (black staining marked by blue arrows), these were shown to stain distinct material, although colocalization was observed in some instances maybe due to binding of bead-bound proteins to the mucus bundles. On the other hand, the *Lotus tetragonolobus* lectin LTL was shown to stain the MUC5B mucin in the submucosal gland as well as in its duct (Fig. 2E). Furthermore, when the 40 nm fluorescent beads were added to the tracheal surface and the mucus bundles were stained with LTL, beads and bundles were not colocalized (Fig. 2F). The transport speed of the 40 nm fluorescent beads was estimated to be about 1 mm/min, whereas the Alcian blue bundles moved significantly slower (0.3 mm/min, Fig. 2G). We have tried to isolate the bead-labeled material separately from the Alcian blue-stained bundles to perform proteomics and identify their nature, but it has not been possible to adequately separate these, as they easily bind to each other. Overall, these results show that the 40 nm fluorescent beads do not stain the mucus bundles and the substrate of their binding remains unclear.

### 3.3 MUC5AC coats the MUC5B bundles

**Fig. 3:**
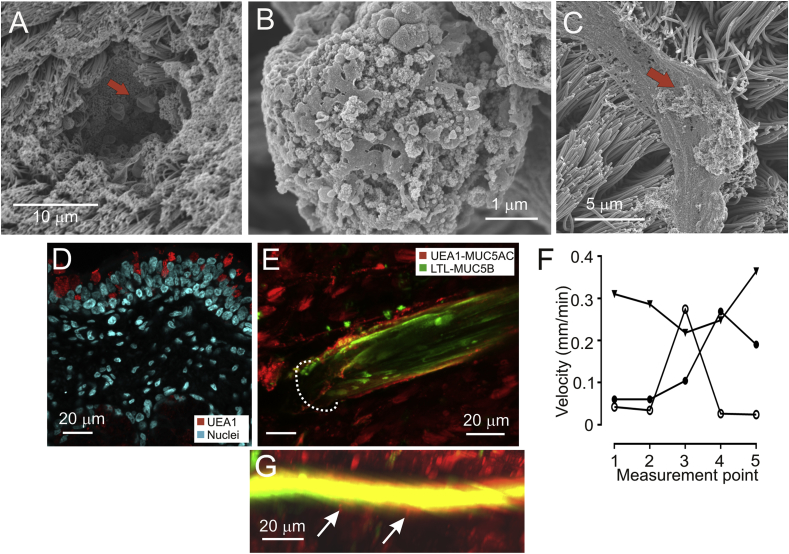
**Mucus bundles have a core of MUC5B and are coated with MUC5AC**. A. Goblet cell in gland opening (red arrow), SEM. B. Close-up of a secreting surface goblet cell. C. Mucus bundle with attached material (red arrow, SEM). D. UEAI lectin (red) stains surface goblet cell mucins (MUC5AC). E. Pig trachea stained with lectins LTL and UEAI. The bundle consists of an LTL-stained core and coated in UEAI-stained material. The dashed line indicates the gland opening. F. Examples of the variable transport speed of Alcian blue-stained mucus bundles measured at five points evenly spaced along each individual bundle in pig trachea (31 bundles from 3 pigs). G. Pig trachea stained with LTL (green, MUC5B) and UEAI (red, MUC5AC) reveals MUC5AC coming from goblet cells (arrows).

To further study why the mucus bundles were moving slower than the beads, SEM micrographs of piglet tracheas were analyzed. When the openings of the submucosal glands were studied, protrusions typical for goblet cells were observed along the terminal part of the gland ducts (Fig. 3A and B) as observed previously [16]. The mucus bundles coming out of the glands and bundles on the surface were covered by lumps of material (Fig. 3C), which was similar to the mucus protruding from the goblet cells, suggesting that the mucus bundles might be partly covered by goblet cell derived material. As described above, when the scraped total mucus and individual mucus bundles were analyzed by absolute quantification both MUC5B and MUC5AC mucins were present (Fig. 2B, Table 1 and Table S1). These facts together could suggest that the lumps covering the MUC5B mucus bundles contained the MUC5AC mucin.

The LTL lectin specifically stained the submucosal gland mucins identically to the *anti*-MUC5B antibody (Fig. 2C and E). The UEAI lectin, on the other hand, stained the surface goblet cells as did the *anti*-MUC5AC antibody (Fig. 3D). When mucus bundles coming out of the submucosal glands were stained with these two lectins and studied by confocal microscopy, a central core of LTL-stained MUC5B was observed patchily covered with UEAI-stained MUC5AC mucin (Fig. 3E).

A major question was why the mucus bundles were not moving as fast as the beads? One possibility was that the bundles were held back in the submucosal glands. The bundle movement was measured at five points evenly spaced along individual mucus bundles, showing that the velocities were very variable and the bundles were not only anchored in one end (Fig. 3F). Another possibility was that the MUC5AC that coated the bundles was attached to the surface goblet cells. A closer study of MUC5B and MUC5AC mucins stained by lectins at the epithelial surface showed that the MUC5AC mucin reached out from the goblet cells onto the MUC5B mucin bundles (Fig. 3G). This could then hold back the bundles and explain the slower speed and uneven movement. The results could suggest that the MUC5AC mucin might connect goblet cells and mucus bundles.

## 4 Discussion

**Fig. 4:**
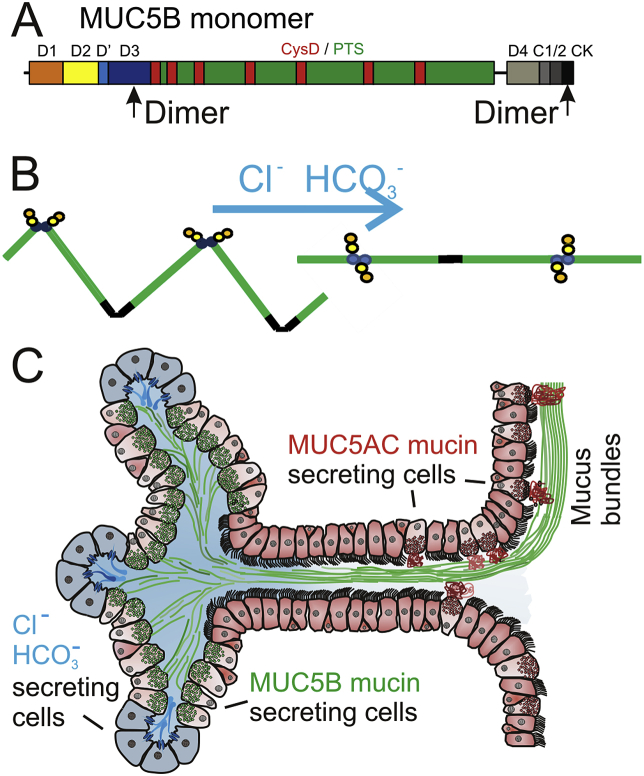
**Model of how submucosal glands shape MUC5B mucin bundles**. A. The MUC5B monomer consists of domains: D1 (orange), D2 (yellow), D' (light blue), D3 (blue) and D4 (light grey), CysD (red), PTS (mucin) domains (green), VWC (C1 (medium grey) and C2 (dark grey)) and CK domain (black). B. Drawing illustrating how the MUC5B mucin is pulled into linear molecules by the Cl^−^ and HCO_3_^−^ fluid flow. C. Schematic drawing of a submucosal gland. The serosal cells at the bottom of the gland are light blue and secrete chloride and bicarbonate. Mucus-secreting cells secrete MUC5B that during transport in the gland form bundles (green). Goblet cells secreting MUC5AC are found in the last gland duct and surface (red).

The MUC5B mucin produced by submucosal glands is forming linear molecules by covalent dimers in both its *N*- and *C*-termini (Fig. 4A) [9]. All mucins are stored packed in the secretory granules of the mucin-producing cells at low pH and high Ca^2+^. Upon release, the mucin is unfolded, a process that requires HCO_3_^−^ to increase the pH and remove the mucin-bound Ca^2+^ (Fig. 4B). In the submucosal glands (Fig. 4C), a bicarbonate and chloride-rich fluid flow is generated by the most peripheral serous cells that have abundant CFTR channels [8], [16]. The fluid flows along the MUC5B mucin secreting cells and this directed flow could unwind the mucins as suggested in Fig. 4B. This unfolding of MUC5B into linear extended bundles is similar to the closely related von Willebrand factor, which is pulled out from the endothelial cells into linear polymers by the blood flow [13]. Appearing at the openings of the glands are bundles with a diameter of 20–30 μm, suggesting that >1000 MUC5B molecules have interacted laterally to form these bundles. This is also similar to what has been observed for the von Willebrand factor [3]. The submucosal glands can, by their organization, be efficient machines for generating thick bundles that are transported by the beating cilia cephalically and ventrally, thereby moving bacteria and debris to the larynx.

In the normal tracheobronchial tree a thin ASL of about 10 μm covers the epithelium, of which about 4 μm is made up by cilia in pigs [2], [16]. The mucus bundles described here are substantially thicker (20–30 μm) when leaving the submucosal glands. The movement of the ASL has been estimated to 3–10 mm/min, a velocity compatible with the fluorescent beads as measured previously and slightly faster than measured here [2], [7], [16]. The Alcian blue-stained mucus bundles, on the other hand, moved considerably slower (0.3 mm/min). Together this suggests that the mucus bundles are floating on and in the ASL, and are transported by the cilia-generated ASL movement. As the mucus bundles do not move as fast as the ASL, they must be retained in some way. Our results suggested that the mucus bundles were anchored on multiple sites on the surface epithelium. Since the mucus bundles were relatively densely coated with the MUC5AC mucin reaching out from the goblet cells, it is likely that the MUC5AC will transiently couple the mucus bundles to the goblet cells. The MUC5AC mucin interaction with the MUC5B mucin bundles could be due to the numerous CysD domains in both these two mucins [1]. Our previous studies of the small intestine show that the small intestinal MUC2 mucin is made anchored to the goblet cells and is detached from these by the protease Meprin β [12]. A similar mechanism may act in the respiratory tract. Having the mucus bundles held down on the epithelial surface is intuitively attractive as this will prevent the mucus bundles to fall off into the lumen and allow control of their movement separately from the cilia beating.
