# Supplementary figures and images for: The normal trachea is cleaned by MUC5B mucin bundles from the submucosal glands coated with the MUC5AC mucin
Source: Biochem Biophys Res Commun. 2017 Oct 21;492(3):331–7. doi: 10.1016/j.bbrc.2017.08.113 (PMC5596833; doi:10.1016/j.bbrc.2017.08.113)

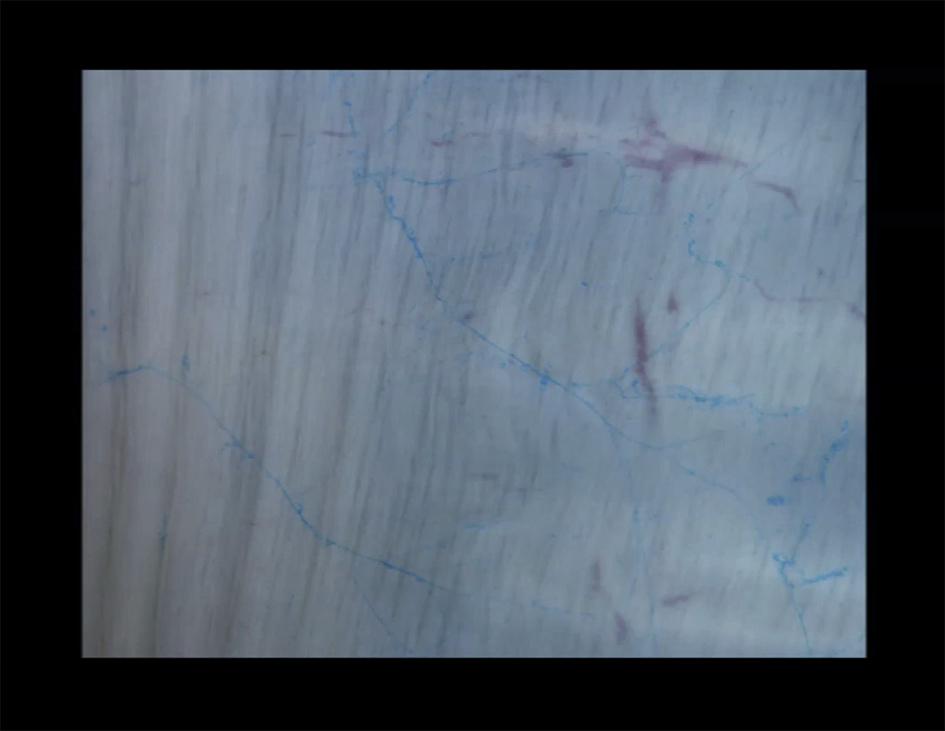

Supplement: Movie S1 — Video of Alcian blue-stained mucus bundle movement. [file mmc1.jpg]
